# Counting CD4^+^ and CD8^+^ T cells in the spleen: a novel *in vivo *method for assessing biomaterial immunotoxicity

**DOI:** 10.1093/rb/rbu003

**Published:** 2014-10-20

**Authors:** Shyh-Jou Shieh, Prashanth Varkey, Po-Yang Chen, Su-Ya Chang, Lynn L.H. Huang

**Affiliations:** ^1^Division of Plastic and Reconstructive Surgery, Department of Surgery, National Cheng Kung University Medical College and Hospital, Tainan, Taiwan, ^2^Research Center of Excellence in Regenerative Medicine, National Cheng Kung University, Tainan, Taiwan, ^3^Advanced Optoelectronic Technology Center, National Cheng Kung University, Tainan, Taiwan, ^4^International Research Center for Wound Repair and Regeneration (iWRR), National Cheng Kung University, Tainan, Taiwan, ^5^Institute of Biotechnology, College of Bioscience and Biotechnology, National Cheng Kung University, Tainan, Taiwan and ^6^Institute of Clinical Medicine, College of Medicine, National Cheng Kung University, Tainan, Taiwan

**Keywords:** cytotoxic lymphocytes, collagen matrix, immunotoxicity, spleen, flow cytometry

## Abstract

As immunotoxicity assessments of newly developed biomaterials are often restricted to use in assessment of local tissue response at the implantation site, they do not always show an immune response acceptable to qualify them for clinical use. We tested a new method to assess systemic toxicity: counting the CD4^+^ and CD8^+^ cells in the spleen. Three different biomaterials were subcutaneously implanted in three groups of rats for the same time period. After 31 days, their spleens were harvested, and CD4^+^ and CD8^+^ cells were counted. The mean CD4^+^/CD8^+^ cell counts were 24.5 ± 3.6/19.8 ± 4.0 (porous collagen matrix group), 25.5 ± 7.1/21.6 ± 3.8 [synthetic collagen matrix (Duragen®) group] and 28.1 ± 4.1/19.6 ± 3.7 (porcine dermis group). Differences in cell counts were not significant. The immunotoxic response generated against porous collagen matrix was comparable to that produced by a similar biomaterial already used clinically. This is, to the best of our knowledge, the first study on cytotoxic lymphocytes in the spleen to quantify systemic immune response to a biomaterial; however, such studies have been conducted with bacterial and viral antigens, and with vaccines. We believe that the present study provides a viable method for larger studies to confirm our current findings.

## Introduction

Evaluating the reaction of the immune system with a foreign antigen is seemingly complex because the immune system that embraces various organs ranges from the peripherally located lymph nodes to the more centrally placed thymus and spleen, gut-associated lymphoid tissue, and interconnecting lymphatic and vascular channels. In addition, the antigenicity of the material being tested as well as its route of administration and dose affect local and systemic immune responses. It is essential to study the immune responses caused by implanting newly developed biomaterials in order to determine their safety for clinical use. Although national health regulatory organizations offer several guidelines for such tests, there are a few unequivocally sufficient and accurate methods for individual biomaterials. This is probably due to the complexity of immune system and the wide variety of new biomaterials being tested. Hence, a battery of tests must be considered for individual biomaterials to obtain an appropriate assessment of their immunotoxicity.

Immunotoxicity refers to any effect on the structure or function of the immune system, or on other systems as a result of immune system dysfunction. An effect is considered to be immunotoxic if it impairs the humoral or cellular immunity needed by the host to defend itself, or if it causes unnecessary tissue damage, such as autoimmunity, chronic inflammation or hypersensitivity. The immune system is adaptive and uses alternative mechanisms to compensate for particular deficiencies in function. This reason forms the basis for suggesting that using *in vivo* animal model tests rather than *in vitro* tests may provide a more accurate picture of immune competence and immunotoxic potential.

*In vitro* tests for cytotoxicity are usually elution tests which compare the effects of contact between monolayer cell cultures and controls with the tested materials. Although most of these studies have used fluid extracts from the experimental biomaterials, an appropriate *in vivo* assessment model is a crucial consideration in the safety evaluation of tissue-engineered products that may contain potential immunoreactive materials. Simple implant studies in which only local tissue reactions are evaluated are not considered systemic toxicity studies, regardless of how long the implants are in place. Immune responses to eliminate foreign particles involve cytotoxic T lymphocytes (CTLs) and non-specific cells such as natural killer (NK) cells and macrophages. Such kinds of effector mechanisms are directed at allogeneic cells, malignant cells, virus-infected cells and chemically conjugated cells. Immune activation of T cells results in a population of effector CTLs, which are often CD8^+^ and, in rare instances, CD4^+^. Resting CTL or precursors, often referred to as CTL-P, are incapable of killing target cells until they are activated. For such activation to happen, a signal is provided when the T-cell receptor and CD8 cells on the CTL-P interact with an antigenic peptide class-I MHC (major histocompatibility complex) complex. A second signal, from cytokines, is induced by activated CD4^+^ T cells. It may be generally assumed that cytokines produced by the activation of CD4^+^ T cells are essential for a complete effector response from CTL. These CD4^+^ T cells, often referred to as T_H_ or T helper cells, are essential for generating CD8^+^ T-cell-mediated target cell lysis. Hence, for a proper correlation of CD8^+^ T-cell counts, a sufficiently large population of CD4^+^ T cells appears necessary. Cytotoxic activity in the spleen has been reiterated by studies on the dynamics of cell activity in mice after a nearly total splenectomy [1]. Several studies have reported experimental evidence that T cells proliferate differentially in lymph nodes and the spleen [2–4]. A comparison of mice that were intravenously infected with vesicular stomatitis virus with mice that were orally given recombinant *Listeria monocytogenes* expressing ovalbumin (*L. mOVA*) showed that, with different antigens and different routes of administration, the activated CD8^+^ cells were dissimilarly distributed in the lymphoreticular system [2]. The CD8^+^ T-cell response in similar studies proved that secondary responses do occur in the spleen [3]. Experiments with influenza virus antigens showed that the proliferation of influenza-specific CD4^+^ and CD8^+^ T cells was predominantly found in the spleen [4].

Antigen-specific CD8^+^ T-cell responses did occur in the spleen with the defined peptides from *L. monocytogenes* and lymphocytic choriomeningitis virus. Most of the studies agree that the nature and route of administration of these antigens affect the immune response in a particular location in the immune system, and that these two factors also influence the fate of T cells, which may proliferate and become effector cells, or else become anergic and die. Dendritic cells are crucial because they are the major population of antigen-presenting cells (APC) in the lymph nodes. The anatomical site of antigen presentation, but not the kind of APC, governs the type of immune response elicited [5].

Our laboratory developed a porous collagen matrix (PCM) from decellularized porcine dermis (PD) [6]. After considering current opinions on the methodology of immunotoxicity evaluation, in the present study, we used the implantation method to examine the systemic immune response to our biomaterial by counting CD4^+^ and CD8^+^ cells in the spleens of Sprague Dawley rats.

## Materials and Methods

All experimental procedures were performed after obtaining approval from the committee on Animal Laboratory Procedures at the School of Medicine, National Cheng Kung University, Tainan, Taiwan. The study was conducted using 15 male Sprague Dawley rats (weight: 250–300 g) randomly assigned to three groups of five each. The rats were maintained in an air-conditioned environment and fed commercial rodent pellet feed and water *ad libitum*. Our test material [6], PCM, was subcutaneously implanted in the PCM group rats, and its immunotoxicity results were compared with unprocessed PD, used in the PD group, and a synthetic collagen matrix [DuraGen® (DG); Integra LifeSciences Corporation, Plainsboro, NJ, USA], in the DG group.

### Implantation procedure

Each rat was anesthetized using an intraperitoneal injection with combination (0.25 ml/100 g of body weight) of atropine (0.1 ml), ketamine hydrochloride (4 ml) (Ketala®; Pfizer, HsinChu, Taiwan), and 2% xylazine hydrochloride (1.34 ml) (Rompun®; Bayer Health Care, Monheim, Germany). After confirming that the rats did not respond to pain, their ventral trunk hair was clipped off. A subcutaneous pocket was created using a 1.25-cm incision. We chose the subcutaneous route based on a study [7] which reported that placing the antigen subcutaneously evoked a satisfactory response in CD4^+^ and CD8^+^ T cells. For each surgical procedure, an implant measuring 1.25 × 1.25 cm was placed in the pocket created on the ventral aspect of the trunk. Serial implants of the same material were placed in non-adjacent pockets on days 0, 14, 19 and 28 of a 31-day experiment. The wounds were closed with nylon sutures. All rats were maintained for a sufficient period (1–3 days) in a warm environment during the recovery period. As such collagen-based materials are weakly immunogenic, we used serial implantation as an antigenic booster to increase the detectability of the immune response.

### Spleen procurement and sample preparation

After 31 days, the spleens were harvested and the animals were killed with an overdose of sodium thiopental. Their spleens were weighed and single suspensions were prepared from them by gently squashing them with a syringe plunger and 10 ml of phosphate-buffered saline (PBS) solution in a Petri dish. The mixture obtained was filtered to obtain single-cell suspensions that were then centrifuged at 300×*g* to collect cell pellets. After the pellets had been fixed, they were resuspended in PBS with 2% benzenesulfonic acid. We made two samples of resuspended cells from each rat’s spleen and separately incubated each for 30 min with an excess of fluorescein isothiocyanate-conjugated mouse anti-rat CD4 monoclonal antibody and PE-anti-rat CD8a (both from BioLegend, San Diego, CA, USA). We identified lymphocyte subsets using a flow cytometer (FACScan; Becton Dickinson, Mountain View, CA, USA). The positive cells were expressed as a percentage of the 10,000 cells counted.

## Results

**Figure 1.**
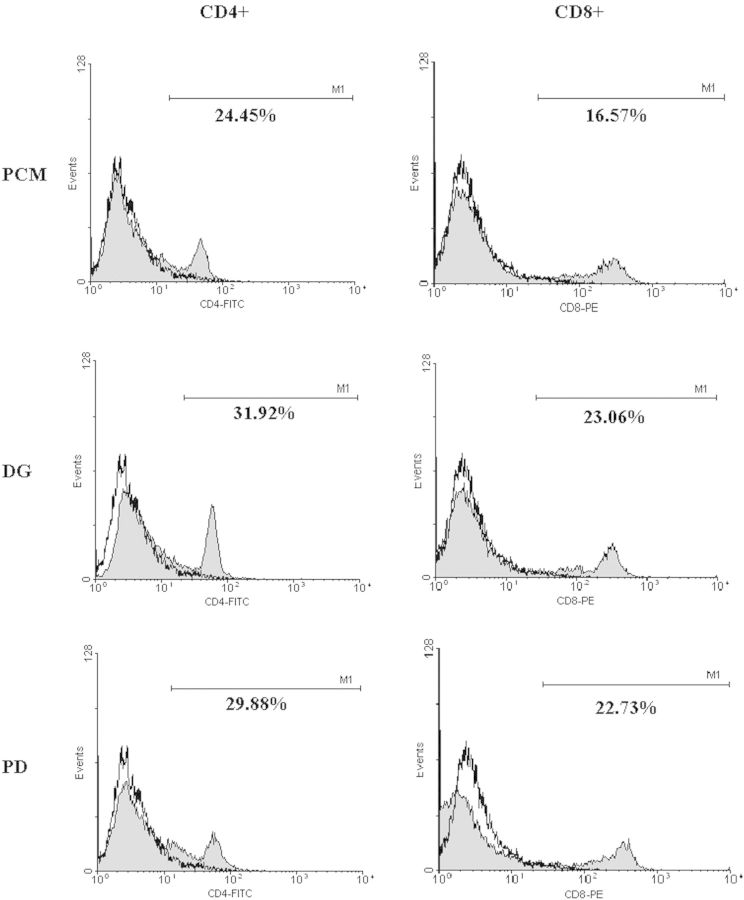
The CD4^+^ and CD8^+^ cell counts calculated by flow cytometry from all three groups in rat number 2.

Figure 1 shows representative data of the flow cytometry from all three groups for CD4^+^ and CD8^+^ cell counts in rat number 2. The mean CD4^+^/CD8^+^ cell counts were 24.5 ± 3.6/19.8 ± 4.0 (PCM group), 25.5 ± 7.1/21.6 ± 3.8 (DG group) and 28.1 ± 4.1/19.6 ± 3.7 (PD group). Total inter-group differences between CD4^+^ and CD8^+^ cell counts were not significant (Table 1).

**Table 1.** Comparative data on CD4^+^ and CD8^+^ cell counts calculated using flow cytometry in the PCM, DG and PD groups

	Percentage of CD4^+^/CD8^+^ cells in different groups					
Rat number	PCM group		DG group		PD group	
	CD4^+^ cells	CD8^+^ cells	CD4^+^ cells	CD8^+^ cells	CD4^+^ cells	CD8^+^ cells
1	24.44	14.7	31.06	17.51	31.21	16.2
2	24.45	16.57	31.92	23.06	29.88	22.73
3	21.78	20.63	28.49	26.31	23.95	18.95
4	21.3	23.73	20.19	17.72	23.22	16.06
5	30.3	23.28	15.77	23.27	32.1	24.05
Mean ± SD	24.5 ± 3.6	19.8 ± 4.0	25.5 ± 7.1	21.6 ± 3.8	28.1 ± 4.1	19.6 ± 3.7

All values are expressed as a percentage of positive cells for the respective marker of 10,000 cells counted.

SD, standard deviation.

### Correlation of spleen weight and cytotoxicity

**Figure 2.**
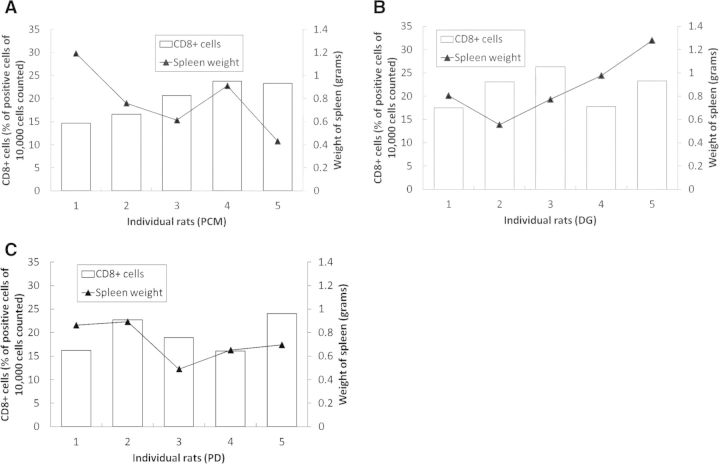
A combination plot with individual sample spleen weights (in grams) plotted as the line and their corresponding CD8^+^ cell counts (expressed as % of positive cells of 10,000 cells counted) plotted as the columns; the plots of the individual groups: (**A**) PCM, (**B**) DG, (**C**) PD show no correlation between the spleen weight and the CD8^+^ cell count, the indicator of cytotoxicity.

Increases in organ weights have been associated with immunotoxicity [8]. The weights of the spleens recorded are summarized in Table 2. The spleen weights of the rats were compared with the corresponding CD8^+^ cell counts, which are the indicator of the cytotoxic cellular response (Fig. 2A–C). Spleen weight and CD8^+^ cell counts were not correlated in any group (Pearson Product-Moment Correlation test).

**Table 2.** Spleen weight of individual rats in the PCM, DG and PD groups

	Spleen weight (in grams)		
Rat number	PCM	DG	PD
1	1.192	0.804	0.862
2	0.760	0.555	0.891
3	0.613	0.772	0.490
4	0.910	0.977	0.650
5	0.430	1.28	0.696

#### Comparison of CD4^+^ cell counts

**Figure 3.**
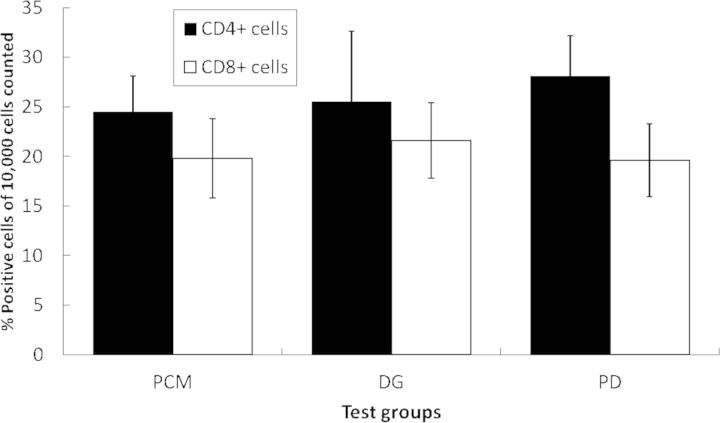
Comparison of CD4^+^ and CD8^+^ cell counts (expressed as % of positive cells of 10,000 cells counted). Note: CD4^+^ and CD8^+^ cell counts were similar in the PCM, DG and PD groups; differences were not significant.

The mean CD4^+^ cell counts were 24.5 ± 3.6 for the PCM group, 25.5 ± 7.1 for the DG group and 28.1 ± 4.2 for the PD group (Fig. 3). Although the absolute values were the highest for the PD group, the differences in the mean values between groups were not significant (one-way analysis of variance (ANOVA)).

#### Comparison of CD8^+^ cell counts

The mean and standard deviation of the CD8^+^ cell counts were 19.8 ± 4.0 for the PCM group, 21.6 ± 3.8 for the DG group and 19.6 ± 3.7 for the PD group (Fig. 3). Although the absolute values of CD8^+^ cell counts were marginally higher for the DG group, one-way ANOVA showed no significant difference between them.

## Discussion

The safety of biomaterials intended for clinical use needs to be established by assessing their immunotoxicity response. International standards organizations such as American Society for Testing and Materials (ASTM) standard or International Organization for Standardization (ISO) have elaborate lists of tests that can be used. The biological response to subcutaneously injected collagenous implants has been histologically assessed by analyzing the tissue around the injected site [9]. The systemic immune response to subcutaneously implanted porcine collagen has been studied by quantifying antibodies in serum [10]. A tiered approach has been suggested as a logical method to choose from among these tests. Evaluating lymphocyte populations in the spleen is one of the tier II tests for evaluating cytotoxicity [11]. The International Conference of Harmonization ICH S8/step 4 document and the European Note for guidance on repeated dose toxicity (HCH 3BS2a, CPMP/SWP/1042/99) also suggest evaluating cytolytic lymphocytes (including CTL and NK cells) [12]. These lymphocytes contain azurophilic granules that accumulate at the site of contact with the target cells and suggest their involvement in cell-mediated killing [13,14]. These granules contain perforin (also called pore forming protein) and cytolysin which are responsible for cell lysis. Perforin is located with CD8^+^ T cells and CD16 cells [15]. Azurophilic granules are absent in inactivated resting T cells. Analyzing CD8^+^ T cell populations may thus seem an optimal method for assessing the cytotoxic response to an implanted biomaterial. Choosing the appropriate tissue for evaluating CD4^+^ and CD8^+^ T cells, however, has been much debated.

Another consideration in our study was the size of the implant to use, because this initial ‘dose of antigen’ may determine the number of CD8^+^ T cells recruited. Contrary to the expectation that smaller doses of antigen can cause a lower response, it has been shown that, although smaller doses recruit fewer T cells, these cells become effector T cells with the ability to produce interferon, and become long-lived memory cells [16,17]. These findings governed our decision to use implants as small as 1.25 × 1.25 cm.

Other considerations in designing our study were adopted from Organization for Economic Cooperation and Development (OECD) Test Guideline 407, which is primarily for testing pharmaceuticals [18]. Repeatedly inserting the same materials in noncontiguous locations was done so that it would act as an antigenic booster. Naïve CTLs become committed as soon as 2 h after they have been exposed to APCs [19]. Subsequent division and differentiation occur without needing further antigenic stimulation of daughter cells, which suggests the autonomous nature of CTL. Compared with this brief stimulatory period for CD8^+^ CTL, about 24 h of continuous sustained signaling is required to prime CD4^+^ CTL [20]. Despite the absence of antigens beyond day 2, T-cell division continues at a constant rate, and the antigen-independent later stage depends on the presence of interleukin (IL)-2. There are contrasting differences in both the duration of antigenic stimuli and the persistence of such stimuli required to optimally activate CTL; hence, we varied the duration of antigenic stimulation by retaining serial implants for 72 hours, 5 days and 14 days, which provided three advantages. First, it allowed for varying periods of antigenic persistence; second, it permitted us to obtain tissue for histopathology analysis at varying periods of time (data not shown); and, finally, repeated placements acted as immunogenic boosters that raised the immune response to detectable levels. CTL responses were neither pronounced nor significantly different between CD4^+^ and CD8^+^ T-cell counts in PCM or DG. Therefore, it may be inferred that the CD4^+^ and CD8^+^ T-cell responses for PCM are comparable to that of DG, a similar collagen matrix already in clinical use. However, conclusive evidence of the benefits of PCM or DG compared with PD will need larger trials and simultaneous comparisons of the cytotoxic responses in animals given sham operations and no biomaterial implants. Such a comparison might also shed light on T-cell response to a surgical wound in the spleen.

Increased organ weight has also been suggested as a method to gauge cytotoxicity [8]. Nevertheless, it is impractical to observe a trend in weight variation following repeated antigenic stimulation over a period of time in the study design. We compared the weight of the spleen at the end of the experiment with the corresponding CD8^+^ cell counts, but found no correlation between spleen weights and CD8^+^ cell counts, the indicator of cytotoxicity.

Individual CD4^+^ cell counts were comparable between the three groups. CD4^+^ cells are important for the CD8 cell response [16,17], which suggests that comparing CD8 cell counts was reliable. The mechanism of effect of CD4 cells was thought to be by providing IL-2, but recent studies [21,22] report that activation of dendritic cells serves as APCs for CD8^+^ T cells, and that it is crucial for differentiating peripheral T cells into T_H_1 and T_H_2 cells [23].

The principal indicator of the degree of cytotoxicity in this study was the CD8^+^ cell counts; there was no significant difference in these counts among PCM, DG and PD. However, their absolute values show a higher CD8^+^ count for PCM than for PD. The explanation for this is that PCM used in test rats was heterogeneous because of minor technical differences in sterilizing and in preparing the biomaterials, which was done by different personnel. Our preliminary results were encouraging; however, because they showed that the *in vivo* immunotoxic response to our newly developed PCM biomaterial was similar to the response to DG, a similar collagen-based material currently in clinical use.

## Conclusion

The present study may be the first attempt to study the systemic immune response by assaying activated lymphocytes in the spleen. It was a preliminary study and requires additional investigation and more convincing data. Standardizing the preparation of materials, including simultaneous comparisons with sham controls (rats without implanted biomaterials), and assessing circulating CD4^+^ and CD8^+^ T cells in peripheral blood are required to obtain confirmatory evidence of the level of toxicity.

## Funding

Department of Technology, Taiwan Ministry of Economics [95-EC-17-A-19-S1-053]; the Taiwan National Science Council [NSC 96-2314-B-006-047, NSC 97-2314-B-006-046-MY2 and NSC 99-2314-B-006-013-MY2].

*Conflict of interest statement.* None declared.
